# Identification of patients who will not achieve seizure remission within 5 years on AEDs

**DOI:** 10.1212/WNL.0000000000006564

**Published:** 2018-11-27

**Authors:** David M. Hughes, Laura J. Bonnett, Gabriela Czanner, Arnošt Komárek, Anthony G. Marson, Marta García-Fiñana

**Affiliations:** From the Departments of Biostatistics (D.M.H., L.J.B., G.C., M.G.-F.) and Molecular and Clinical Pharmacology (A.G.M.), Institute of Translational Medicine, and Department of Eye and Vision Science (G.C.), Institute of Ageing & Chronic Disease, University of Liverpool, UK; and Department of Probability and Mathematical Statistics (A.K.), Faculty of Mathematics and Physics, Charles University, Prague, Czech Republic.

## Abstract

**Objective:**

To identify people with epilepsy who will not achieve a 12-month seizure remission within 5 years of starting treatment.

**Methods:**

The Standard and New Antiepileptic Drug (SANAD) study is the largest prospective study in patients with epilepsy to date. We applied a recently developed multivariable approach to the SANAD dataset that takes into account not only baseline covariates describing a patient's history before diagnosis but also follow-up data as predictor variables.

**Results:**

Changes in number of seizures and treatment history were the most informative time-dependent predictors and were associated with history of neurologic insult, epilepsy type, age at start of treatment, sex, and having a first-degree relative with epilepsy. Our model classified 95% of patients. Of those classified, 95% of patients observed not to achieve remission at 5 years were correctly classified (95% confidence interval [CI] 89.5%–100%), with 51% identified by 3 years and 90% within 4 years of follow-up. Ninety-seven percent (95% CI 93.3%–98.8%) of patients observed to achieve a remission within 5 years were correctly classified. Of those predicted not to achieve remission, 76% (95% CI 58.5%–88.2%) truly did not achieve remission (positive predictive value). The predictive model achieved similar accuracy levels via external validation in 2 independent United Kingdom–based datasets.

**Conclusion:**

Our approach generates up-to-date predictions of the patient's risk of not achieving seizure remission whenever new clinical information becomes available that could influence patient counseling and management decisions.

Epilepsy is a heterogeneous disorder with respect to etiology, seizures types, and outcome. Although for most patients seizures can be controlled with antiepileptic drugs (AEDs), ≈30% never enter a sustained remission from seizures, despite multiple treatment changes. Patients typically undergo years of treatment before the clinician is confident that their epilepsy is drug resistant.^1,2^ Uncontrolled seizures during this time can have a pronounced reduction in quality of life, education, and employment prospects. The ability to reliably predict earlier that seizure remission will not occur would offer the opportunity for more effective management and better patient counseling in an attempt to minimize adverse effects on quality of life.

The focus of this study is to predict patients who will not achieve a 12-month remission by 5 years of follow-up using covariates collected at baseline and during follow-up. Several studies have assessed prognostic factors for outcomes in newly diagnosed epilepsy,^3–10^ but prediction of drug-resistant epilepsy based on baseline (or early follow-up) prognostic factors has achieved areas under curves of only 61% to 78%. This work is different because we use data collected during follow-up (referred to as longitudinal variables) in addition to baseline covariates that describe a patient's history before diagnosis. Our approach generates up-to-date predictions of the patient's risk of not achieving seizure remission whenever new clinical information becomes available, providing a framework to aid decision making.

## Methods

### Patients and procedures

The Standard and New Antiepileptic Drugs (SANAD) trial^11,12^ is a randomized controlled trial that recruited 2,437 people with epilepsy assigned to 1 of 2 arms of the trial. Arm A included those for whom carbamazepine was considered the first-line standard treatment, primarily patients with focal epilepsy, who were randomized to treatment with carbamazepine, gabapentin, lamotrigine, oxcarbazepine, or topiramate. Arm B included those for whom sodium valproate was considered the first-line standard treatment, primarily those with generalized or unclassified epilepsy, who were randomized to lamotrigine, topiramate, or valproate. Clinicians recruiting patients into SANAD were primarily neurologists with expertise in epilepsy. This dataset contains a large, heterogeneous group of patients with epilepsy, many of whom have been observed for at least 5 years, and provides the opportunity to investigate the individual profiles of patients to predict those who will not achieve a 12-month continuous seizure remission during follow-up. Our multivariate analysis includes data from both arms of the SANAD study simultaneously. Previous analysis of the SANAD data used only baseline covariates to identify prognostic factors influencing a patient's time to remission from seizures or treatment failure, and the development of a clinical classification tool was not addressed.^13,14^

This analysis considers 1,577 patients who achieved at least 1 continuous period of 12-month remission within 5 years from starting treatment and 175 patients who did not. Patients were included in our analysis if they had experienced at least 2 clinically definite unprovoked seizures, were at least 5 years old, and had been followed up either to first remission or for 5 years. Our aim was to correctly identify patients who did not achieve remission as early as possible in their treatment journey by looking at the individual profiles (using both baseline and longitudinal data), and our primary outcome was a binary variable indicating whether the patient achieves remission within 5 years of starting treatment.

For the purposes of external validation, the Multicentre Study of Early Epilepsy and Single Seizures (MESS)^15^ and National General Practice Study of Epilepsy (NGPSE)^16^ datasets were used, which come from a United Kingdom–based randomized controlled trial and the UK primary care system, respectively.

### Standard protocol approvals, registrations, and patient consents

SANAD received appropriate multicenter and local ethics and research committee approvals and was managed according to the Medical Research Council's Good Clinical Practice Guidelines. Patients gave informed written consent to inclusion and to long-term follow-up. SANAD is registered as an International Standard Randomised Controlled Trial (No. ISRCTN38354748).^17^

### Predictive models and follow-up data

Two models are fitted, one for the remission group and one for the no-remission group. These models are then used to predict for a new patient the likelihood of not achieving remission by assessing which of the 2 models the new patient's profile is closer to. Predictions are updated each time new information becomes available for a patient. The model does not need to be refitted, and the new patient's data are used to generate a patient-specific prediction of the risk of not achieving remission.

In this study, 4 follow-up variables were considered for inclusion in the final model based on clinical consensus. These were (1) whether a patient experienced seizures (of any type) since their last clinic visit, (2) the total number of seizures experienced since the last clinic visit, (3) whether treatment was changed at the last visit, and (4) the number of adverse events experienced by the patient since the last visit. Common adverse events experienced included depression, dizziness, allergic reactions, headaches, tiredness, pins and needles, and weight gain.^18^ Treatment change could include the addition or removal of an AED or a change in the dose of a drug.

The list of potential baseline covariates considered included age at the start of treatment, sex, type of epilepsy, EEG results, CT/MRI results, first-degree relative with epilepsy, neurologic insult, and total number of seizures experienced before treatment was started. Patients were classified as having a neurologic insult if they had learning disabilities or a neurologic deficit. Epilepsy type was classified as focal, generalized, or unclassified and is highly correlated with the randomization arm to which the patient was recruited. EEG and CT/MRI results were defined as normal, not clinically indicated, or abnormal. Previous analysis of the SANAD dataset used these baseline covariates to identify prognostic factors influencing a patient's time to remission.^13^ In this analysis, they are used to model the evolution over time of the longitudinal variables that predict no-remission and only indirectly influence the classification procedure through their influence on the longitudinal variables.

Models also accounted for time since starting treatment and for time since the last follow-up to account for the fact that clinic visits were not equally spaced.

### Statistical methods

Multivariate generalized linear mixed modeling was applied to model the longitudinal variables in each prognostic group separately (patients who achieved 12-month remission and patients who did not).^19^ Multivariate models account for the correlation between repeated measurements over time and for the dependence of the longitudinal variables on baseline covariates.

Multivariate models were built by first considering the subset of baseline covariates that best described the changes over time of each of the 4 longitudinal variables listed above. Models were compared by use of penalized expected deviance^20^ alongside a forward selection approach. Penalized expected deviance is a loss function that penalizes for model complexity and is suitable for complex hierarchical models. These models were then used to assess the probability that new patients would not achieve remission within 5 years of starting treatment in a discriminant analysis. The best combination of longitudinal variables was determined by probability of correct classification. Training sets consisting of data from 70% of patients in each group were used to build the model, and data from the remaining 30% were used to test the model. Training and test sets were randomly generated 100 times, and the results were averaged.

### Dynamic classification scheme

To classify patients as remission or no remission, we used the following procedure. First, the risk of not achieving remission for each patient was predicted with the multivariable model. Second, we stated a threshold for the predictive risk. The threshold chosen in this work is 0.64, which was associated with the point on the receiver operator characteristic curve nearest to the top left corner (i.e., it provides the best balance in terms of number of patients correctly identified as no remission and those correctly identified as 12-month seizure-free patients). Third, the uncertainty of the risks predicted by the model varies across patients (and over time). To account for this uncertainty, credible intervals (bayesian equivalent of confidence intervals [CIs], an interval in which we are 99% confident that the true probability of not achieving remission during 5 years lies) were used to assess the confidence in the assigned risk.^21^

We applied the following allocation scheme:

1. We consider the first visit of a patient and calculate both the probability of the patient not to achieve remission during 5 years after starting treatment and a 99% credible interval around this probability.
2. If the credible interval is entirely above the threshold of 0.64, we assign the patient to the no-remission group. Prediction now stops for this patient because the patient shows a high risk of not achieving remission.
3. If the credible interval is entirely below 0.64, we temporarily assign the patient to the remission group and update the patient's risk at the next visit.
4. If the credible interval contains 0.64, the patient remains unclassified (because of the level of uncertainty in the estimated risk, the patient is not yet assigned to a group). Their risk is updated at the next visit.

### Data availability

Anonymized data used in this study are available on request from Dr. Marson.

## Results

**Figure 1:**
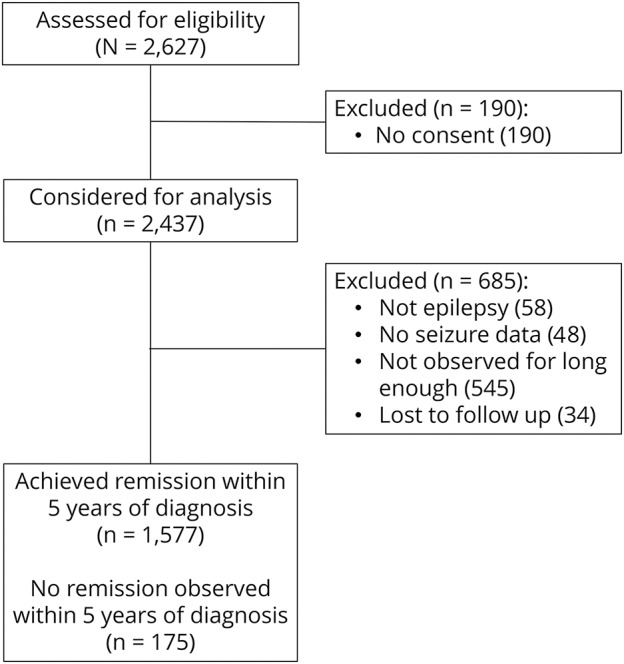
Standard and New Antiepileptic Drugs trial profile

Figure 1 shows the disposition of patients recruited to SANAD who were included in the analyses reported here. Table 1 shows their baseline variables.

**Table 1:**
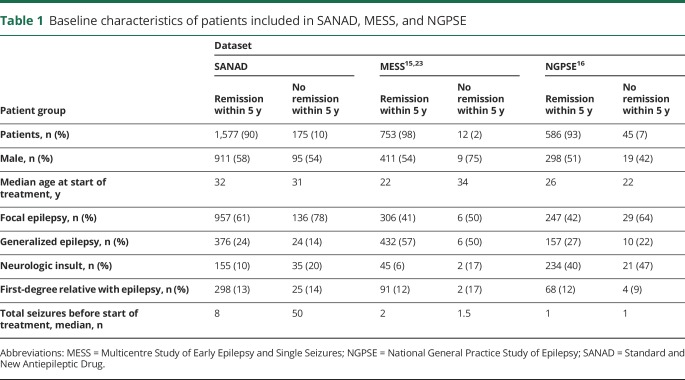
Baseline characteristics of patients included in SANAD, MESS, and NGPSE

The best multivariate models consisted of 2 longitudinal variables: whether the patient experienced seizures since the last clinic visit and whether the patient's treatment was changed at the last clinic visit. The baseline covariates included in each model, along with their odds ratios, are described in table 2.

**Table 2:**
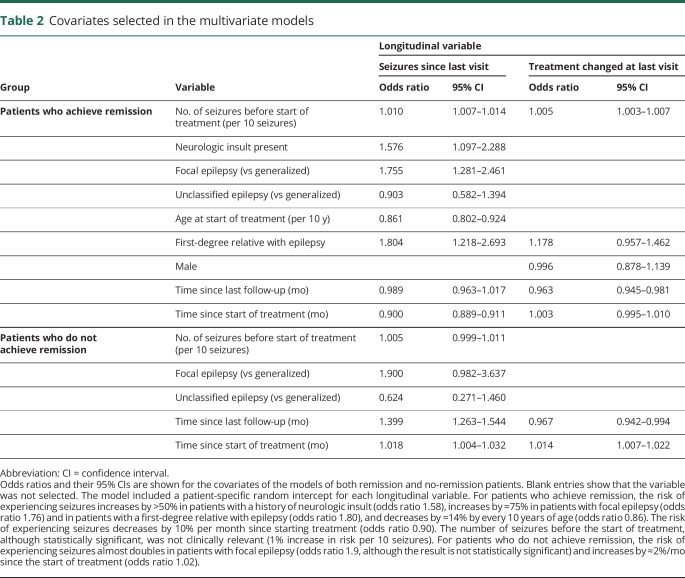
Covariates selected in the multivariate models

Having focal epilepsy increases the risk of experiencing seizures, regardless of whether the patient will ultimately experience remission (table 2). Having a first-degree relative with epilepsy or neurologic insult increases the risk for patients who will ultimately achieve remission. These variables were not selected in the model for patients who do not achieve remission, although this could be due to smaller sample size.

**Figure 2:**
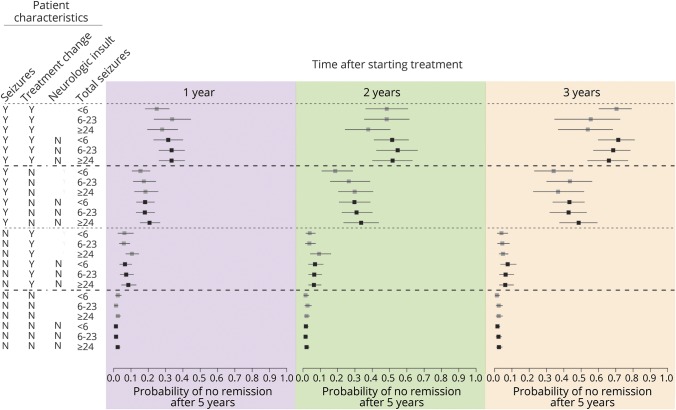
Probability of no remission within 5 years of starting treatment for combinations of risk factors at 3 chosen time points Box represents the point estimate average of the predicted probability of not being in remission for patients with the given characteristics; line represents the 99% credible interval. Seizures and treatment change columns refer to the period since the last clinic visit, while neurologic insult and total seizures are recorded at baseline. The risks are calculated at 1, 2, and 3 years after starting treatment.

Figure 2 describes how the assigned probability of not achieving remission changes according to follow-up factors. Patients who experienced seizures or whose treatment was changed since the previous visit tend to be assigned higher probabilities of not achieving remission. These assigned probabilities increase as the length of observation increases for a patient. In general, patients with a history of neurologic insult are assigned lower probabilities of ultimately being no remission compared to patients with similar seizure and treatment history but without neurologic insult, especially early in their observation history (e.g., 1 year), reflecting the fact that patients with neurologic insult are more likely to experience seizures,^13^ at least initially, regardless of whether they will ultimately achieve remission.

**Figure 3:**
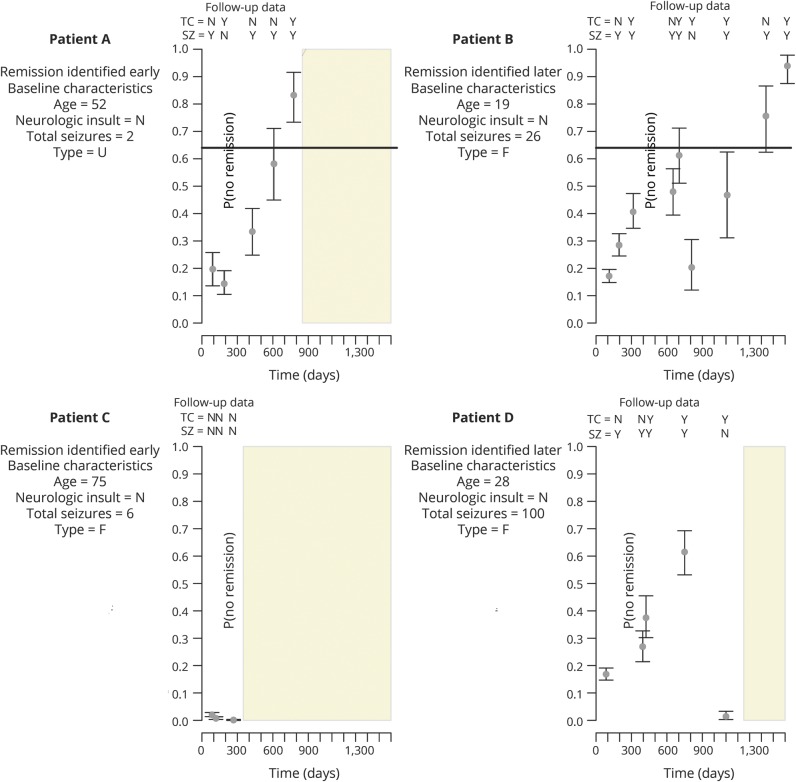
Profiles of risk for 2 no-remission patients correctly classified 2 years (patient A) and just over 4 years (patient B) after starting treatment and for 2 patients with remission correctly classified soon (patient C) and ≈3 years (patient D) after starting treatment Points represent the probability assigned to the patient, while the bars show the 99% credible interval around each probability of no remission. The observations recorded along the top of each plot indicate whether the patient experienced seizures (SZ) since the last visit and whether treatment was changed at last visit (TC); the information on the left of each plot describes some baseline characteristics for each patients. Light yellow bars show the selected threshold of 0.64. Gray box shows the remaining period of the 5 years since starting treatment once a patient's status has been predicted. F = focal; U = unclassified.

Figure 3 illustrates the allocation scheme for 4 real scenarios. Patient A initially has a low probability of not achieving remission because, despite having experienced seizures, the time of observation is still short. At the next visit, the risk drops because a treatment change led to a short period free from seizures. As more information is gathered on the patient, the patient’s probability of not achieving remission increases, but only after the fifth visit is the entire 99% credible interval above the chosen threshold of 0.64. At this point, we are more confident this patient is not going to achieve remission because the patient has changed treatment 3 times and is still experiencing seizures. This patient is classified as no remission within 2 years, a much earlier time point than the decision might otherwise be made in clinical practice.

Patient B takes longer to be classified as no remission. After 2 years, the patient changed treatment, and it resulted in a period without seizures, which caused the assigned probability of not achieving remission to decrease dramatically, offering hope that this patient may achieve remission. Unfortunately for this patient, the seizures returned, causing a steady increase in the probability of being in the no-remission group, until the patient is confidently predicted to be no remission after just over 4 years.

Patient C achieves remission immediately after 12 months. This is accurately predicted by the model because the patient experiences no seizures after the start of treatment and the clinicians felt no need to change treatments. In contrast, patient D experienced seizures for a longer period. That patient had focal epilepsy, had a high number of seizures before starting treatment, and was relatively young, which meant that although the patient continued experiencing seizures for >2 years, the risk of not achieving remission was not initially high because seizures were likely to occur according to these baseline characteristics. Just over 3 years after starting treatment, the patient is observed to have changed to a treatment that appears to give adequate seizure control and is correctly classified as remission.

The overall accuracy of our predictive model is assessed by considering how many patients from the test sample were correctly classified. With the use of the selected threshold of 0.64 and the exclusion of patients left unclassified by the model, 95% of patients who truly did not achieve remission were identified as such (sensitivity, 95% CI 89.6%–100%), while 97% of patients who achieved remission were correctly classified (specificity, 95% CI 93.3%–98.8%). Overall, 97% (95% CI 93.4%–98.5%) of patients were correctly classified. For patients predicted by the model as not achieving remission, 76% (95% CI 58.5%–88.2%) truly did not achieve remission (positive predictive value [PPV]). The area under the receiver operator characteristic curve (AUC) was 94.5% (95% CI 90.9.0%–96.7%).

A randomly chosen split of the data into training and test sets achieved a calibration slope of 0.922, suggesting that the model is well calibrated (calibration slopes close to 1 show well-calibrated models with observed and expected risks matching well), in addition to providing good discrimination between patients who achieve remission and those who do not.^22^

Five percent of patients were left unclassified, and including these patients, the model achieved an overall sensitivity of 78% and specificity of 93% with a probability of correct classification of 91%.

The average time at which a patient was correctly identified as not achieving remission was 36.4 months (just over 3 years). In fact, 51% of patients who were correctly identified as not achieving remission were identified within 3 years, while only 10% required a visit in their fifth year after starting treatment to be correctly classified (table 3). For most patients who will not achieve remission, our model can identify them between 1 and 3 years. Our model is patient specific. The classification of no remission is not made at the same time for all patients (unlike the previously published predictive models^8–10^) but only when their individual risk is high enough.

**Table 3:**
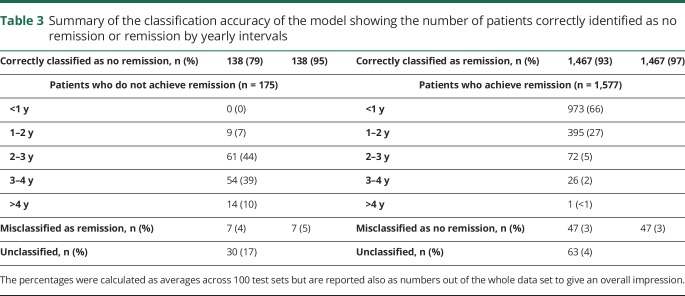
Summary of the classification accuracy of the model showing the number of patients correctly identified as no remission or remission by yearly intervals

With the use of the dynamic classification scheme with 99% credible intervals, only 5% of patients were left unclassified and would require further follow-up in clinical practice to determine their classification. The time of prediction is less important for patients who achieve remission because these patients would in practice remain under observations until remission is observed. Nevertheless, 973 of 1,577 patients (62%) who achieve remission can be correctly identified with only observations from their first year of follow-up.

### External validation

We conducted an external validation study of our predictive model using 2 additional datasets: MESS^15,23^ and NGPSE (table 1 for descriptive details).^16^ MESS was a United Kingdom–based randomized controlled trial comparing immediate and deferred treatment for patients who experienced a first unprovoked seizure or had epilepsy. NGPSE is an unselected cohort from the UK primary care system of people with newly diagnosed seizures. We used the model built using all the SANAD data to predict the status of patients in MESS and NGPSE and assessed the accuracy of the predictions. In the NGPSE data, all counts of ≥11 seizures were recorded as >10. This affected only a relatively small number of patients (9 of 45 no-remission patients and 57 of 586 remission patients). Following previous examples,^24^ we used various missing data imputation methods to assign values >10 for the total number of seizures before starting treatment to the relevant patients in NGPSE. The different imputation methods used gave almost identical results, so only the hot deck imputation is reported here.

The accuracy in terms of sensitivity and specificity of the classified patients when the predictive model is applied to MESS and NGPSE is high (table 4, i.e., sensitivity >90% and specificity >95% for classified patients, only 1% of patients left unclassified) and comparable to the prediction accuracy within SANAD (obtained by splitting the data into training and test sets). In both the MESS and NGPSE cohorts, very high AUC values demonstrate excellent model discrimination. For the MESS data set, the PPV was lower, although we suspect this is due to the low proportion of patients who do not achieve remission.

**Table 4:**
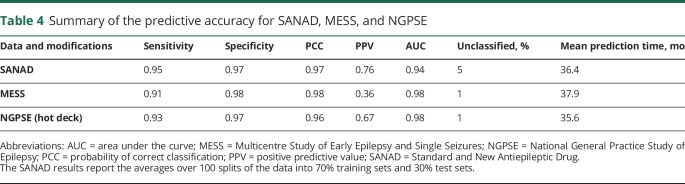
Summary of the predictive accuracy for SANAD, MESS, and NGPSE

The NGPSE cohort was well calibrated, achieving a calibration slope of 0.95, suggesting that the SANAD model is well calibrated and discriminates well even in this external dataset. The MESS cohort achieved, on the other hand, a calibration slope of 0.52. Calibration slopes in external datasets are often worse than in the dataset used to train the model. In the case of the MESS cohort, the worse calibration slope is likely also to be influenced by the low proportion of patients who do not achieve remission, as was seen in the PPV value for MESS.

This external validation shows that the SANAD model was able to correctly identify people with epilepsy who would not achieve remission from seizures within 5 years of beginning treatment with a high degree of accuracy.

Differences are nevertheless expected because the datasets are not identical in purpose or form but can be considered to come from the same “superpopulation” as required for external validation.^22^

## Discussion

Our dynamic prediction approach allows the identification of patients who will not achieve remission within 5 years of starting treatment. The time at which nonremission is identified is different among patients, reflecting the heterogeneous nature of epilepsy and patient trajectories. For classified patients, 95% of no-remission cases are correctly identified, and the majority of these patients are identified within the first 3 years after beginning treatment. Some patients are clearly missed by remaining unclassified, and the overall sensitivity would be reduced to 78% by taking into account unclassified patients.

The most informative evidence in terms of predicting a patient's epilepsy status is the combination of treatment changes and seizure history together with baseline factors that accurately predicts a patient's prognosis. Note that these results do not imply that changing treatment causes an increased likelihood of not achieving remission because changing treatment has been shown to usually be beneficial.^25^ Instead, the fact that a clinician felt the need to change the dose or type of the AED at the previous visit is used as an indicator that something was not going well.

While clinicians may at first be concerned about choice of drug and dosing and the effect that choice might have on prognosis, it is important to consider that randomized controlled trials in epilepsy have largely failed to find important differences among AEDs. This was exemplified by the use of noninferiority designs in the European Union and historical controlled withdrawal to monotherapy trials in the United States. In contrast, we have clearly demonstrated the effect of clinical factors on outcome.

The International League Against Epilepsy proposed a definition of drug-resistant epilepsy as the failure of adequate trials of 2 tolerated and appropriately chosen and used AED schedules (whether as monotherapies or in combination) to achieve sustained seizure freedom.^2^ This definition, however, is of little use in clinical practice (e.g., “adequate” and “appropriately chosen” are subject to interpretation, and the choice of 2 AEDs is arbitrary), and patients currently wait years before clinicians are confident that they will not achieve remission. In contrast, our analysis includes dose and drug changes, thereby using information about treatment in a more quantifiable and informative way, avoiding any need to make judgments about adequacy and appropriateness.

In our dataset, a total of 600 (of 1752) patients (34%) stopped taking their randomized drug. However, 413 of these 600 patients were changed to a different drug, and 187 received no AEDs (of whom 159 [85%] achieved remission).

Patients who did not achieve remission from seizures within 5 years of starting treatment had an average of 5.2 treatments changes (either a change in dose or the addition or removal of an AED) over the duration of the 5 years for which they were observed. Patients who were correctly identified as not achieving remission by our model had undergone an average of 3.9 treatment changes at the point at which they were correctly identified. The International League Against Epilepsy definition of drug-resistance requires only 2 changes in treatment to classify a patient as drug resistant. Our analysis suggests that, in practice, more treatment changes occur before we can be confident a patient will not achieve remission.

Previous models have defined remission/no remission differently from us, so a direct comparison is not possible. However, in the prediction of terminal remission at 5 years after diagnosis using baseline characteristics,^8^ a sensitivity of 65%, a specificity of 64%, a PPV of 36%, an NPV of 85%, and an AUC of 0.7 were achieved. When the prediction used data collected during a 6-month follow-up period,^8^ these values increased slightly to 69%, 71%, 43%, 88%, and 77%, respectively. A model that was built to predict drug resistance after 10 years (based on values at 1 year of follow-up) reported AUCs between 61% and 76%.^10^ Finally, a model developed to predict 2-year status after 6 months of follow-up reports an AUC of 77.97%.^9^ The predictive accuracies from our model reported above are higher than these values (even when sensitivity/specificity accounts for the unclassified patients).

The optimal threshold should be selected according to the clinical objective. A higher threshold will produce greater specificity and greater PPV, but a longer time is required on average to identify patients who will not achieve remission. Conversely, a lower threshold will reduce the time required to identify patients who will not achieve remission; the sensitivity of the test will increase but at the cost of a reduction in specificity.

The baseline characteristics influence the time at which a patient's status can be confidently predicted, while seizure and treatment history have the largest influence on the predicted status. This discovery is illustrated by comparing the first visits of patients A and B in figure 3. Patient B has characteristics at the start of treatment that are associated with an increased likelihood of experiencing seizures for at least some time, whereas patient A has characteristics that would not lead a clinician to expect that the patient would experience seizures. Their first visits occur at approximately the same time, and they both have experienced seizures and are still on the first treatment assigned. However, at baseline, patient A is assigned a probability of not achieving remission that is 5% higher compared to that of patient B because it is expected that patient B experienced seizures. Because patient A continues to experience seizures, the estimated risk that the patient will ultimately not achieve remission increases much more quickly than for patient B.

A limitation of the model is that patients who achieve remission are considered only up to the point at which they first achieve remission. In the SANAD data, 532 of 1,577 patients who were observed to achieve a 12-month remission went on to have further seizures (34%) while under observation. Of these, 183 (34%) experienced 1 further seizure only, and 236 of 532 patients (44%) were observed to achieve 12-month remission again.

A number of patients with insufficient follow-up time to determine their 5-year status were excluded from the analysis (n = 545, mostly administrative censoring). In addition, 34 patients dropped out of the study before it was possible to determine their status; therefore, it was not possible for these patients to be included in the analysis. The omission of this group of patients may have an influence on the model parameters for each multivariate generalized linear mixed model and hence also on the discriminant analysis. However, given that this exclusion is dictated mainly by the time of starting treatment, we do not expect that this led to biased results.

Although a group of patients have been excluded from this analysis, the proportion of patients achieving a 12-month remission within 5 years of follow-up is comparable to that in previous studies, suggesting that the included cohort is reasonably representative of people with epilepsy. Previous estimates suggest that 60% to 70% of people will achieve remission from seizure, defined as a 5-year period of continuous remission within 9 years of follow-up.^26^ Table 1 in that study reports that after 5 years of follow-up, 93% (91%–95%) of patients had achieved a 12-month remission interval, which is comparable to the 90% observed in this study.

Although possible, we do not expect that the imputation of the number of seizures in the NGPSE dataset has dramatically increased the degree of agreement with SANAD given that this was applied to only a relatively small number of patients (10%).

This is a step toward a clinical tool for identifying patients who will not achieve remission from seizures. The model has been internally and externally validated, and it has the potential to be used in clinical practice to aid the stratification of patient management and to influence patient counseling.

Patients who will not achieve remission can be identified by recording at baseline (at the beginning of treatment with AEDs) the following information: patient's age, sex, type of epilepsy, number of seizures experienced before treatment, whether the patient had a first-degree relative with epilepsy, and whether the patient had a neurologic insult, as well as by monitoring over time whether the patient had experienced seizures since the last clinic visit and whether the clinician had felt it necessary to change treatment at the last visit. Because a small group of patients are unclassified, increased confidence is obtained in the predictions of those patients who are classified as not likely to achieve remission. This selection of baseline and longitudinal variables, along with the times of the clinic visits after baseline, is sufficient to classify patients with high levels of accuracy.

## Publication history

Received by *Neurology* December 15, 2017. Accepted in final form August 15, 2018.

## Glossary

AED: antiepileptic drug
AUC: area under the receiver operator characteristic curve
CI: confidence interval
MESS: Multicentre Study of Early Epilepsy and Single Seizures
NGPSE: National General Practice Study of Epilepsy
PPV: positive predictive value
SANAD: Standard and New Antiepileptic Drug
